# Updates and New Perspectives on Adenoviral Gene Therapy and Vaccine Vectors

**DOI:** 10.3390/v15020514

**Published:** 2023-02-13

**Authors:** Thomas Dobner, Luca D. Bertzbach

**Affiliations:** Department of Viral Transformation, Leibniz Institute of Virology (LIV), 20251 Hamburg, Germany

Adenoviruses are commonly used as efficient high-capacity vectors and excellent gene delivery vehicles. Their applications range from basic molecular research to gene therapy and recombinant viral vector vaccines. Adenoviral vectors are currently used in regenerative and cancer therapies, and as first-generation COVID-19 vaccines. Despite their widespread use and constant progress, various challenges and safety concerns still limit the application of adenoviral vectors to their fullest potential. Our Special Issue, “New Aspects of Adenoviral Vaccine Vectors and Adenoviral Gene Therapy”, presents five excellent original research articles and reviews on adenoviral vector development, recent trends in adenovirus-mediated oncolytic therapies, and lessons learned from current large-scale adenoviral vector vaccination programs.

An in-depth review article by Erwan Sallard and colleagues provides the latest insights into adenoviral vector design and reiterates lessons learned from adenovirus-based COVID-19 vaccines [1]. Their review also provides a summary of vector-induced side effects and discusses how they can be avoided by improving rational vector development.

A research article by Xiang Du and colleagues summarizes an in vivo proof of concept for a replication-competent adenovirus vector vaccine against a morbillivirus of veterinary importance [2]. They show that upon oral administration, the vaccine induces neutralizing antibodies; this is important information, especially in the context of wildlife vaccination in veterinary medicine.

Nora Bahlmann and colleagues contribute new data on improved adenovirus vectors for oncolytic and gene therapies [3]. Their research article characterizes vector candidates (JO-4 vectors) with enhanced carcinoma-specific transduction capacity.

A thorough review by Wen-Chien Wang and colleagues [4] recapitulates the importance of pre-existing anti-vector and transgene immunity and, thus, the activation of innate responses in adenoviral vector-based therapies. Moreover, they outline approaches to dealing with these issues to improve vector efficiency.

Paola Blanchette and Jose Teodoro comprehensively review the characteristics of oncolytic adenoviruses that are currently undergoing clinical trials for cancer therapy [5]. Their overview discusses the combination of oncolytic adenoviruses with immune checkpoint inhibitors, as well as common mutational modifications in the viral genome. They correctly state that although research on human adenoviruses has spanned many years, the ways in which these viruses interact with the immune system remain poorly understood. These important aspects of virus–host interactions must be improved.

This collection of articles provides valuable new insights into various properties of adenoviral vectors in vaccine and gene therapy settings—a topic that has been studied for decades but is more relevant than ever, as there have never been so many individuals injected with a recombinant adenovirus vector.
